# A multidimensional perspective on microbial interactions

**DOI:** 10.1093/femsle/fnz125

**Published:** 2019-06-11

**Authors:** Alan R Pacheco, Daniel Segrè

**Affiliations:** 1 Graduate Program in Bioinformatics and Biological Design Center, Boston University, 24 Cummington Mall, Boston, MA, 02215, USA; 2 Department of Biomedical Engineering, Department of Biology and Department of Physics, Boston University, 24 Cummington Mall, Boston, MA, 02215, USA

**Keywords:** microbial interactions, microbial communities, mutualism, microbiome, microbial ecology, systems biology of metabolism

## Abstract

Beyond being simply positive or negative, beneficial or inhibitory, microbial interactions can involve a diverse set of mechanisms, dependencies and dynamical properties. These more nuanced features have been described in great detail for some specific types of interactions, (e.g. pairwise metabolic cross-feeding, quorum sensing or antibiotic killing), often with the use of quantitative measurements and insight derived from modeling. With a growing understanding of the composition and dynamics of complex microbial communities for human health and other applications, we face the challenge of integrating information about these different interactions into comprehensive quantitative frameworks. Here, we review the literature on a wide set of microbial interactions, and explore the potential value of a formal categorization based on multidimensional vectors of attributes. We propose that such an encoding can facilitate systematic, direct comparisons of interaction mechanisms and dependencies, and we discuss the relevance of an atlas of interactions for future modeling and rational design efforts.

## Teaser

Reports of inter-species interactions in microbial communities contain abundant information that defies simple ecological categorizations. Vectors of attributes can formally capture this knowledge, helping create interaction catalogues and predictive models.

## INTRODUCTION

Microbes form complex ecosystems comprising up to hundreds or thousands of different species (Whitman, Coleman and Wiebe 1998; Ley *et al*. 2006; Walker and Pace 2007; Wilhelm and Matteson 2008; Brown *et al*. 2009; Qin *et al*. 2010; Welch and Huse 2011; Tecon and Or 2017). Increased exploration of these communities enabled by new technologies has yielded a wealth of information on their constituent organisms, as well as growing insight into the complex and rich web of relationships the organisms form between each other. As the study of microbial communities embraces a more systems-oriented approach (Raes and Bork 2008; Klitgord and Segrè 2011; Faust and Raes 2012; Fuhrman, Cram and Needham 2015; Poudel *et al*. 2016), more and more attention is being directed toward the myriad ways microbes interact, as well as toward the crucial roles these interactions play in defining community function. These relationships, which can range from mutualistic exchange of metabolic products (Imachi *et al*. 2000; Harcombe 2010), to antagonistic secretion of antibiotics (Jousset, Scheu and Bonkowski 2008; Cornforth and Foster 2015; Kelsic *et al*. 2015), to direct predation of individual organisms (Jurkevitch 2007; Chen *et al*. 2011; Kadouri *et al*. 2013), make up a vast space of relationships that are ubiquitous in the microbial world.

**Figure 1.**
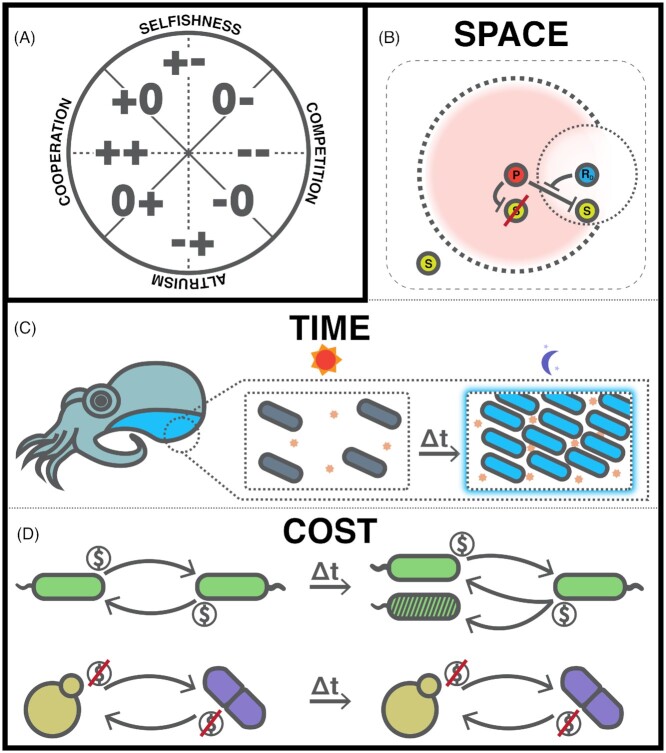
The multifaceted nature of microbial interactions. **(A)**, Axes commonly used to classify interactions, adapted from Lidicker 1979. A single interaction can be represented as a point within the axes, which quantify the ecological outcomes experienced by the participants and the strength of the interaction. For example, a point close to the bottom of the axes (corresponding to −+) represents an altruistic scenario in which one participant experiences a net negative outcome and the second participant receives a positive one. **(B-D)**, Examples of attributes observed in interactions that resist straightforward, benefit-oriented classification. Each of the interactions displayed feature some kind of mutualistic outcome, but exhibit crucial dependencies that impact the nature of the interaction. (B), An interaction that confers differing benefits on its participants based on their spatial configuration, reported by Kelsic *et al*. (Kelsic *et al*. 2015). Here, a colony of *Streptomyces* “P” produces an antibiotic that kills sensitive *E. coli* “S” within a given radius. If, however, an antibiotic-degrading *Streptomyces* population “R_D_” is placed within this radius, *E. coli* is able to survive within its immediate vicinity. Therefore, depending on its location, *E. coli* can either experience a neutral or negative effect from the antibiotic-producing *Streptomyces*. (C), Time-dependent intraspecies interaction between *Vibrio fischeri* cells within the light organ of the Hawaiian bobtail squid. During the day, the squid releases the majority of *V. fischeri*, diminishing their concentration within the organ. As the *V. fischeri* population regrows, individuals secrete signaling molecules that, upon reaching a critical concentration, lead to the expression of luminescence genes. In this way, the symbionts allow the squid to bioluminesce at night. The day–night cycle therefore drives this transition through its effects on squid physiology, signaling molecule concentration, and bacterial cell density. (D), Two mutualistic interactions that impose differing metabolic costs on participants. *Top:* Intraspecies interactions within *Pseudomonas fluorescens* populations reported by Rainey and Rainey (Rainey and Rainey 2003). Initially, cooperating individuals secrete an adhesive polymer to form a biofilm. This process occurs at a metabolic cost to individual organisms. Over time, defecting individuals stopped producing the polymer but continued benefitting from the collective production within the group. This ‘cheating’ diminished the viability of the community in the short term, leading to more complex interaction dynamics over longer timescales. *Bottom:* A simplified schematic of a mutualistic interaction based on non-costly overflow metabolism demonstrated by Ponomarova *et al*. Here, *Saccharomyces cerevisiae* uses overflow metabolism to secrete amino acids which allow for the growth of *Lactococcus lactis*. *Lactococcus lactis*, in turn, provides glucose and galactose to the yeast through lactose hydrolysis, yielding a stable symbiotic relationship (Ponomarova *et al*. 2017). Though these two mutualisms are fundamentally different, neither represents the sole possible outcome of costly or non-costly interactions. Previous work has shown how cheating could in fact stabilize mutualisms (Foster and Kokko 2006), and that cheating itself may pose less of a threat to community collapse as commonly thought (Frederickson 2017).

Despite the diversity and abundance of microbial life on the planet, we still face fundamental challenges in addressing broad, important questions pertaining to microbial interrelationships: are specific types of interactions more common among certain taxa than others? How prevalent is mutualism across all environments? How can we systematically compare interactions across individual studies? A first step in answering these questions is to categorize interactions based on common, recurrent properties. We may start with a widely-used classification system that is based on determining the ecological outcome each organism experiences in a pairwise interaction (Lidicker 1979; Keddy 2001) (Fig. 1A). These outcomes, on a very general level, can be either positive, neutral or negative and can encompass all possible pairs of effects: from mutualism (in which both participants experience a positive outcome) to competition (both participants experience a negative outcome). Between these two extremes are combinations of positive, neutral and negative outcomes such as amensalism, in which the actor experiences no benefit or detriment and the recipient experiences a negative outcome. Though this framework has formed the basis for a broad corpus of ecology research (Faust and Raes 2012), it is limited in that it cannot capture many nuances that are crucial in determining how individual interactions arise, change and affect a community (Margulis 1990; Smith 2001; Martin and Schwab 2013). Moreover, there exist inconsistencies in the very language used to describe these ecological outcomes, hindering comparison of intermicrobial behaviors between different disciplines and prompting efforts to standardize how interactions are described (West, Griffin and Gardner 2007; Smith *et al*. 2019; Tipton, Darcy and Hynson 2019).

In light of these limitations, we ask if it is possible to use additional properties to enhance the vocabulary we use to describe and compare interactions. It has indeed become possible to capture more and more fine details of interactions through recently-developed technologies and computational tools, such as measurements of individual exchanged molecules using metabolomics (Tang 2011; Chamoun, Aliferis and Jabaji 2015; Bilyk and Luzhetskyy 2016), inference of entire co-occurrence networks based on metagenomic sequencing (Qin *et al*. 2010; Steele *et al*. 2011; Barberán *et al*. 2012; Gilbert *et al*. 2012; Lloyd-Price *et al*. 2017), and direct detection of interspecies synergy using microfluidics (Park *et al*. 2011; Hsu *et al*. 2019). Unfortunately, as this wealth of data is often reported on an observation-by-observation basis, it remains difficult to comprehensively classify interactions and systematically compare them across studies. As a way of facing this challenge, we may therefore look for inspiration in integrative categorizations of complex phenomena in other areas of biology (e.g. metabolic networks (Kanehisa and Goto 2000; Caspi *et al*. 2016), protein interaction networks (Szklarczyk *et al*. 2017), or 16s sequences (DeSantis *et al*. 2006)), which have become valuable standardized repositories of knowledge, as well as starting points for data-driven analyses that would have been otherwise impossible (Barberán *et al*. 2012; Magnúsdóttir *et al*. 2016; Goldford *et al*. 2018).

In this mini-review, we compile a diverse list of known microbial interactions and comment on the limitations of describing and classifying them based solely on simple ecological outcomes. We then provide an example of how one can instead embrace the multifaceted nature of microbial relationships by explicitly ascribing numerical variables to different properties of the interacting partners. In doing so, we propose a framework for encoding interaction data in a way that enables quantitative analysis and comparison across studies. This formalization could be viewed as one of many incremental steps needed to better understand these interactions, as well as a way to encourage conversations on how to structure future methodical comparisons, data-mining efforts, and data-driven analyses.

## MICROBIAL INTERACTIONS ARE MULTIDIMENSIONAL, DYNAMIC PHENOMENA

Examples of interactions that can be best understood by more explicitly accounting for different attributes include those that change significantly based on spatial configurations. An interesting example is that of *Aggregatibacter actinomycetemcomitans* (*Aa*) and *Streptococcus gordonii* (*Sg*), bacteria isolated from the human oral cavity. *Sg* has been shown to secrete lactate, the preferred carbon source of *Aa*, as a metabolic byproduct (Brown and Whiteley 2007; Tong, Zeng and Burne 2011). Lactate is taken up by *Aa*, which experiences growth benefits as a result (Ramsey, Rumbaugh and Whiteley 2011). It has been shown in a murine infection model, however, that while this metabolic exchange benefits the virulence capabilities of *Aa*, *Sg* also secretes high amounts of hydrogen peroxide, a potent antimicrobial. Stacy *et al*. demonstrated that *Aa* can adopt a twofold detoxification-dispersion response to this challenge, which allows it to spatially position itself at an optimal distance from *Sg* and detoxify the hydrogen peroxide to consume lactate (Stacy *et al*. 2014). This response is also thought to yield benefits to *Sg*, enhancing the overall fitness of the community and the strength of the infection. This interaction could be termed mutualistic using a simple interpretation of ecological outcome since, despite a clear antagonistic action by one of its participants, it results in a net beneficial relationship. However, only adopting such an interpretation risks abstracting away key nuances within the interaction that detail paradoxical, yet important, mechanisms for cooperation and competition. A different example of spatially dependent interactions was investigated by Kelsic *et al*. using a synthetic community made up of *Escherichia coli* and two *Streptomyces* strains isolated from soil (Kelsic *et al*. 2015). In this study, *E. coli* were exposed to an antibiotic-producing *Streptomyces* strain on an agar plate. As the *E. coli* population was sensitive to the antibiotic produced, it was only able to grow outside the radius formed by the antagonistic *Streptomyces*. However, if an antibiotic-degrading *Streptomyces* strain was placed within this killing radius, *E. coli* were shown to grow in an area around the detoxifying strain (Fig. 1B). As such, the spatial configuration of the interaction directly determined the growth outcomes of its participants. Recent modeling efforts have also demonstrated how the nature of an interaction can change when spatial information is considered (Armitage and Jones 2019), underscoring the importance of capturing these dependencies.

As with spatial organization, time-dependent sharing of nutrients or toxin sequestration has also been shown to affect the nature of microbial interactions. For example, in the same study mentioned above, Kelsic *et al*. used dynamical modeling to show how four organisms with varying degrees of antibiotic production and degradation capabilities could stably coexist in various temporal modes (stable equilibrium, limit cycles, or chaotic oscillations) without spatial separation (Kelsic *et al*. 2015). In nature, the importance of temporal dynamics on interactions is particularly evident in host-microbe symbioses, where circadian cycles impact gene regulation and metabolic processes in the host and among their symbionts (Roden and Ingle 2009; Liang, Bushman and FitzGerald 2015; Heath-Heckman 2016; Staley *et al*. 2017). A classic example of such a scenario is the intraspecies signaling patterns shown within the *Vibrio fischeri* communities living within the squid *Euprymna scolopes*. *V. fischeri* colonize the light organ of the squid, allowing it to bioluminesce at night. This result arises from a sequence of biological processes, all ultimately driven by the day–night cycle (Fig. 1C) (Verma and Miyashiro 2013). As the nature of these interactions vary drastically over different temporal scales, it is important to consider this attribute as a crucial factor in classification.

The classic axes commonly used to describe ecological interactions (Fig. 1A) are also limited in the sense that specific points on this graph (e.g. ++) group together interactions that may be based on very different mechanisms and costs of metabolite production and exchange. For example, two organisms that benefit metabolically from each other (thus falling in the ++ category) may do so in two fundamentally different ways: a first possibility is an interaction mediated by evolved secretion of metabolically costly products (Harcombe 2010; Wintermute and Silver 2010; Celiker and Gore 2012; Mee *et al*. 2014; Zomorrodi and Segrè 2017). A second type of interaction could be the outcome of secreted byproducts that are not costly to the producer, and that are not ‘intended’ to specifically benefit any other particular organism. Such non-costly secretions are also described as byproduct benefits (Sachs *et al*. 2004), and can lead to the emergence of mutually beneficial interactions through selfish actions by individual organisms. Examples of this type of metabolite-mediated exchange include overflow metabolism and secretion of incompletely reduced carbon sources (Molenaar *et al*. 2009; Basan *et al*. 2015), which are secretions that can be strongly dependent on environmental context (Klitgord *et al*. 2010; Chamberlain, Bronstein and Rudgers 2014; Pacheco, Moel and Segrè 2019). Importantly, the cost of metabolite production and secretion can change the interaction's susceptibility to cheating phenotypes (Hamilton 1964; West *et al*. 2006; D'Souza *et al*. 2018), which in turn can have implications for the long-term stability of the relationship (Fig.   1D). Additionally, the strong environmental dependence of interactions is especially problematic when studying ‘uncultivable’ organisms or when designing synthetic microbial communities, whose members may have metabolic dependencies that are difficult to satisfy experimentally (Rappé and Giovannoni 2003; Vartoukian, Palmer and Wade 2010; Stewart 2012). The formation of an interaction often requires the fulfillment of particular environmental conditions, such as the presence of specific carbon or nitrogen sources or a particular pH range, which might be strongly modulated by spatial or temporal dynamics.

For some interactions, the ecological outcomes experienced by their participants remain unclear. Nonetheless, other properties can be clearly identified to yield insight into the mechanisms of the relationship, as exemplified by the interaction between two archaea: *Igniococcus hospitalis* and *Nanoarchaeum equitans*. Both species are able to form a stable co-culture, but such a partnership is only necessary for the survival of *N. equitans*, and not for that of *I. hospitalis* (Huber *et al*. 2003). In co-culture experiments, *N. equitans* appears to rely on H_2_S, the primary metabolic end product of *I. hospitalis*, as well as on amino acids and lipids provided by *I. hospitalis* (Jahn *et al*. 2004, 2008). This interaction would therefore appear to be parasitic. However, this relationship results in no detriment to the *I. hospitalis* population and there even appear to be evolved structural features that allow for a tight physical connection between both organisms. This strong coupling suggests that there is some benefit given by *N. equitans* to *I. hospitalis*, though experiments have so far been unable to identify such a mechanism, making any definitive labeling of an interaction type elusive. This interaction exhibits other important properties, such as contact-dependence, that are not captured in a framework based solely on ecological outcomes.

Finally, interactions that involve three or more species present further classification challenges. It is often possible to assign clear benefits when the participants in a multispecies interaction can be divided into two distinct roles, as in, for example, an interaction observed between *Myxococcus xanthus* and a consortium of prey bacteria (Berleman *et al*. 2006). Here, the predatory *M. xanthus* can be assigned a positive outcome and its prey bacteria can collectively be assigned a negative, detrimental one. Additionally, if an interaction confers effects of the same sign to all recipients (e.g. a tripartite symbiosis in which all organisms benefit (Lőrincz *et al*. 2010) it is possible to place it on an ecological outcome axis (Fig. 1A). If, however, different members within a higher-order interaction are influenced by effects of different signs, as is likely to occur within complex communities, it becomes less clear how to classify the interaction without reducing it to a set of pairwise interactions. This limitation has important implications for understanding the structure and function of natural microbial ecosystems. Though pairwise relationships can be used to gain insights into overall community features (Guo and Boedicker 2016; Friedman, Higgins and Gore 2017; Venturelli *et al*. 2018), their predictive power can vary depending on the model used or on interaction mechanisms (Carrara *et al*. 2015; Momeni, Xie and Shou 2017). As such, the way in which features of higher-order interactions are represented must be carefully considered.

## A MULTI-DIMENSIONAL FRAMEWORK FOR DESCRIBING MICROBIAL INTERACTIONS

The above examples pose the question of whether it is possible to move beyond individual narratives and formally encode multiple features of observed microbial interactions, extending the classical ecological outcome axis. If some of these features are ubiquitous across microbial communities, one could formalize the multidimensional nature of microbial relationships and devise an expanded list of attributes that can classify interactions at a higher resolution and in a manner that better highlights their diverse, dynamic qualities. In this section, we introduce a possible scheme for capturing some of these attributes, and exemplify the application of this framework to a compendium of interactions found in the literature. The attributes we will consider here are: the molecular vehicle (if present) that mediates the interaction, whether the interaction is specific to a particular recipient, whether there is a fitness cost to engaging in the interaction, the site (cytoplasm, membrane or extracellular) in which the interaction takes place, the biome or habitat in which the interaction has been observed, whether the interaction was observed to depend on specific spatial configurations, temporal dynamics or direct physical contact, as well as the sign of the ultimate ecological outcome incurred by the participants. It is our hope that incorporating these attributes, further formalized in Table 1, into descriptions of interactions will enhance our understanding of the landscape of known microbial relationships.

**Table 1.** Definitions of key interaction attributes. We describe microbial interactions using a number of attributes, each of which is assigned a numerical value based on experimental observations. Each attribute defined here corresponds to a column in our interaction catalog (Supplementary Table 1). We quantify most features in a binary way: using a ‘0’ if the interaction does not exhibit a certain attribute and a ‘1’ if it does. For example, if an interaction involved the exchange of a peptide, it would contain a ‘1’ in the ‘peptides’ column. Costs and ecological outcomes are specific to the organisms in the interactions, that is, there are columns for costs and outcomes for each of the participants. In a pairwise commensal interaction, for instance, there would be a ‘0’ in the column corresponding to the outcome gained by participant 1 and a ‘1’ in the column corresponding to the outcome gained by participant 2.

Attribute	Definition	Quantification
Specificity	The reported mechanism of interaction is deployed in a manner specific to the recipient (e.g. signaling molecules specific to one species vs. nonspecific secretion of waste products).	Binary
Cost	Engagement in the reported interaction (e.g. secreting a metabolite) imposes a fitness burden on a participant (i.e. the individual fitness/growth rate of an organism would initially have been greater had it not been involved in the interaction).	Binary
Ecological outcome	The ultimate ecological effect the interaction confers on each participant. Combining these values for both participants in a pairwise interaction yields its overall ecological outcome (e.g. 1,-1 corresponds to selfishness; 1,1 corresponds to mutualism, etc.).	1: Beneficial 0: Neutral −1: Detrimental
Contact dependence	Reported interaction features organisms engaging in direct physical contact.	Binary
Time dependence	Reported relationship features organisms interacting according specific temporal frames (e.g. occurring only at one point in a circadian cycle).	Binary
Spatial dependence	Reported interaction features organisms displaying particular spatial configurations (e.g. colonies separated by some distance on an agar plate as opposed to interacting in mixed cultures).	Binary
Site	The site, relative to the microbes involved, in which the interaction is reported to take place: extracellular (e.g. signaling molecule release or metabolic exchange), membrane (e.g. protein docking or conjugation), or cytoplasm (e.g. direct predation).	Binary value for each site
Habitat	The biome(s) in which the interaction or participating organisms have been observed: aquatic, biofilm, food product, multicellular host, soil, synthetic, or ubiquitous.	Binary value for each habitat
Compounds involved	The type of molecule that mediates the interaction: small molecules (e.g. carbohydrates or metabolic intermediates, but not secondary metabolites), nucleic acids (e.g. DNA), peptides (e.g. amino acids), or secondary metabolites (e.g. quorum sensing molecules).	Binary value for each compound type

The set of interactions to which we applied the proposed framework consists of 74 microbial relationships sourced from the literature, which together make up a ‘catalog’ in which each interaction is characterized by a ‘barcode’ of quantifiable attributes (Table S1, Supporting Information). As we focused almost exclusively on collecting interactions that had sufficient data to assign values to each of our attributes, our catalog is limited to a small set of well-characterized observations. Nonetheless, it is intended to represent a cross-section of all known interactions, featuring a wide variety of biomes, mechanisms, taxa and dependencies. We also note that our assignment of features is specific to the experiments reported in each study. As a result, the attributes we report here should not be considered universal to all interactions between the reported taxa. Instead, they serve to encode the complex nature of observed microbial interactions in a manner that is compatible with current methods of data collection. Nonetheless, the gradual establishment of a standardized set of attributes to be measured in newly designed experiments could lead to large compendia of these multi-dimensional traits that may enable more generalizable insights.

A majority of the interactions that we compiled occurred between two distinct microbial species. However, we have also included some interactions that have been studied at lower taxonomic resolutions (e.g. at the genus or phylum level). Some of these relationships list multiple species of a particular genus — or even relatively undefined consortia of organisms – as individual participants. Although different species within a large taxonomic grouping will undoubtedly display varying interaction properties individually, studies may group several organisms together to highlight their collective performance of a function of interest. For example, a set of nitrogen-fixing archaea was framed as a single entity that engages in an interaction with organisms of the bacterial genus *Desulfosarcina* (catalog entry 6, (Dekas, Poretsky and Orphan 2009)). In grouping these varied organisms together, Dekas *et al*. highlighted the cooperative behavior of the archaeal consortium as it applied to its bacterial symbionts. Since identifying relationships of interest in complex natural microbial systems sometimes necessitates such levels of abstraction, our catalog reflects the taxonomic groupings for each interaction as they were reported.

As an example of how a specific interaction can be translated into our proposed multi-dimensional classification, we will analyze here in detail a predatory relationship between two opportunistic pathogens: *Pseudomonas aeruginosa* and the filamentous form of the fungus *Candida albicans*, initially identified by Hogan and Kolter ((Hogan and Kolter 2002), catalog entry 48). Here, *P. aeruginosa* is reported to form a biofilm on the filaments of *C. albicans*, which kills the fungus. By eliminating a competitor, this action allows *P. aeruginosa* to more effectively consume nutrients. We quantify the ecological outcomes of this interaction by assigning a positive outcome to *P. aeruginosa* and a negative one to *C. albicans*. The study also described mechanisms displayed by *P. aeruginosa*, that were necessary to initiate the interaction, chief among which was an ability to differentiate the filamentous form of the fungus from the yeast form. We therefore deem this interaction to be targeted towards a particular organism and phenotype, as the bacterium did not attack yeast-form *C. albicans*. The authors described a dependence of the interaction on quorum sensing pathways, indicating the probable role of secondary metabolites. Since these molecules are exchanged between organisms, we record the extracellular environment as the primary site for this relationship. As the activation pathways that lead to the formation of biofilms and the secretion of secondary metabolites are metabolically costly, we assign to *P. aeruginosa* a positive metabolic cost of initiating predation. Since the interaction results in the death of *C. albicans*, we also deem the interaction to impose a fitness cost on the fungus. Lastly, the interaction features direct contact between both species and depends on their spatial proximity, so we list contact and space as key dependencies. In all, we describe this interaction with 22 different variables, which collectively make up its interaction ‘barcode.’

Though this ‘barcoding’ constitutes a rudimentary formalization of the complex attributes an interaction can exhibit, it has some immediate ramifications. Firstly, it allows for identification of interaction attributes that may not be intuitively correlated. For example, are interactions involving signaling molecules more likely than others to also exhibit spatial dependence? In which environments could such correlations hold? Using our limited dataset, for instance, we calculated a Spearman correlation coefficient of 0.41 between the column detailing spatial dependence and the column containing secondary metabolite data (compared to −0.24, −0.04 and −0.04 for small molecules, nucleic acids and peptides respectively). Though modest, this significant correlation (*P* = 0.0003) hints at possible relationships between spatial dependence and secondary metabolite use for interactions in our dataset. By performing similar analyses on a greater number of interactions, it may be possible to infer stronger, additional correlation patterns emerging from the data. For example, it would be possible to ask if all ‘barcode’ combinations tend to occur, or if there are specific attribute combinations that are either very frequent or rare. Moreover, it would be possible to highlight the discovery of new types of interactions that exhibit unique attribute combinations not previously observed. Such a framework could also be useful for identifying commonalities among interactions from different biomes, and for comparing seemingly unrelated interactions to each other.

An ability to answer the general questions discussed above would undoubtedly require more data than the small collection of interactions we have compiled here. Moreover, annotating newly discovered interactions to the level of detail we propose is not a trivial task, as generating a single interaction ‘barcode’ likely requires deployment of a variety of different experimental and computational methods (Raes and Bork 2008; Faust and Raes 2012) in addition to manual curation of the catalog itself. Because of these challenges, some mechanistic attributes remain unknown even for the relatively well-characterized set of interactions that we have compiled here. For instance, the presence and strength of a microbial interaction can also be determined by environmental constraints such as temperature (Price and Sowers 2004; Dell, Pawar and Savage 2014; Lin *et al*. 2016) and pH (López-Lara, Sohlenkamp and Geiger 2003; Jin and Kirk 2018; Ratzke and Gore 2018), as well as by the chemical composition of the environments (Klitgord *et al*. 2010; Chamberlain, Bronstein and Rudgers 2014; Ponomarova *et al*. 2017; Ziesack *et al*. 2018; Pacheco, Moel and Segrè 2019). Moreover, microbial interactions may change based on properties of the populations themselves such as relative population size (Kong *et al*. 2018; Venturelli *et al*. 2018), order of colonization (Mazumdar, Amar and Segrè 2013; Dang and Lovell 2016), or stochasticity in community assembly (Zhou *et al*. 2013; Vega and Gore 2017; von Bronk *et al*. 2017). Future cataloguing and analysis efforts would benefit from the reporting of as many of these attributes as possible in individual studies, as well as from improved data mining methods to incorporate existing data into numerical frameworks.

**Figure 2.**
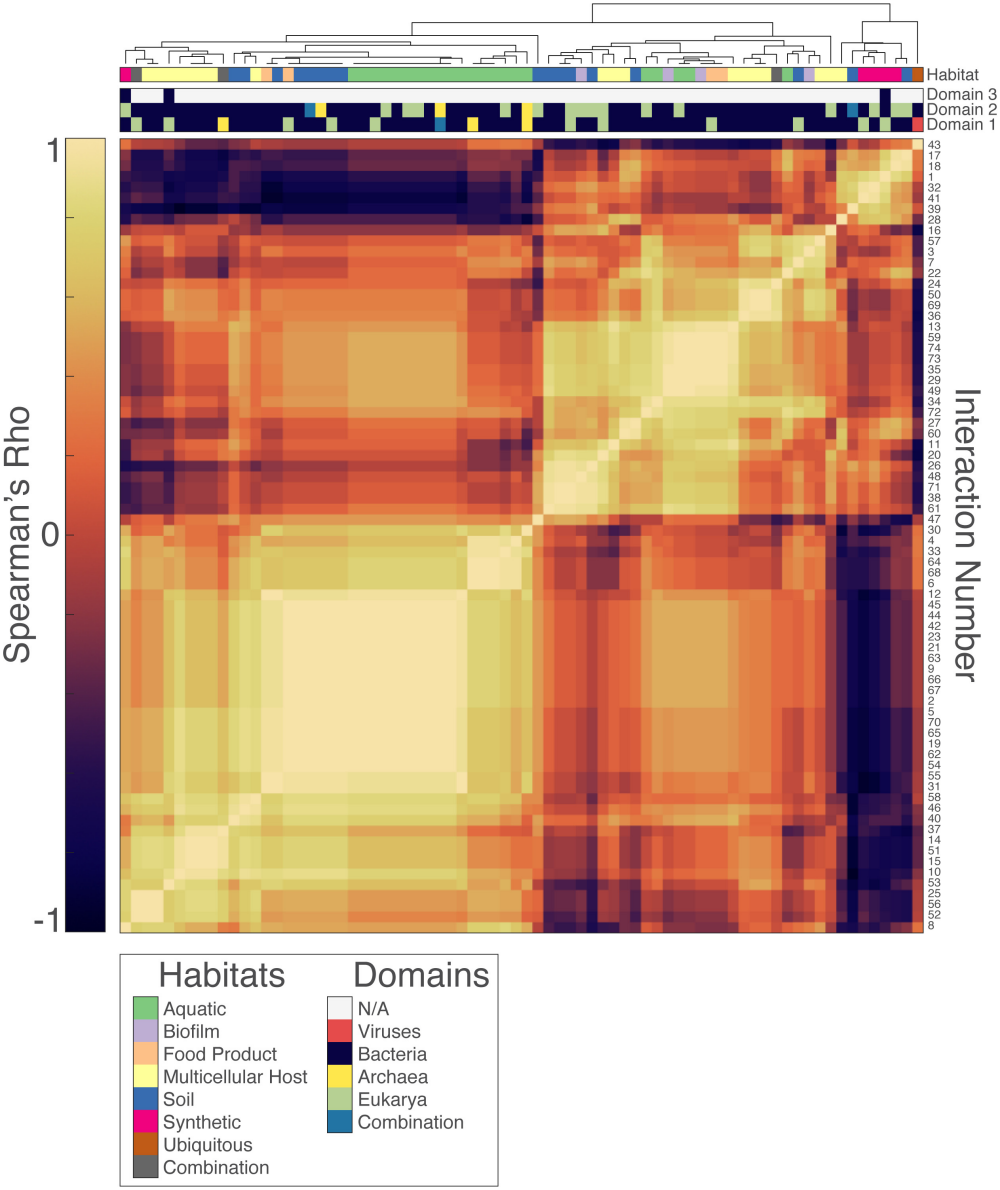
Hierarchical clustering of microbial interactions, numbered according to their catalog entry in Table S1 (Supporting Information). Numerical values for interaction attributes (specificity, costs, ecological outcomes, dependencies, site, habitat and compounds involved) were normalized from 0 to 1. Multi-column attributes (i.e. those that contain specific values for individual participants, such as ecological outcome and cost) were additionally encoded into single unique values using the Cantor pairing function. Unknown values (comprising 2.1% of the dataset) were imputed with the mean of each column to enable all interactions to be compared. The normalized values were used to calculate pairwise distances between each interaction using Spearman's rho. Hierarchical clustering was then performed to generate a tree based on the resulting distance matrix. Taxonomic information (which was not used to generate clustering), as well as habitat information is displayed for all interactions.

Despite these challenges, we will outline how a quantitative method of comparing interactions may be approached using the dataset we have compiled. Here, we used our annotation framework to calculate distance metrics between each interaction. These distances consider, in an unbiased way, all of the attributes we have annotated for each interaction. In this manner, we are able to generate a hierarchical tree and heatmap (Fig. 2) to visualize which interactions are most similar to each other. As our encoding of interaction properties yielded numerical vectors that are difficult to parse and intuitively compare by eye, such a tree (which likely bears no resemblance to evolutionary or phylogenetic trees) is a useful way to visually interpret similarities or differences between interactions. Our clustering yielded several key observations: for example, the case of prophage-bacteria mutualism reported by Barondess and Beckwith ((Barondess and Beckwith 1995), catalog entry 43) is, perhaps intuitively, grouped furthest away from all other organisms. This grouping occurred independently of the large relative taxonomic difference between viruses and bacteria, archaea and eukaryotes, as taxonomic information was not considered for the clustering. Instead, its grouping is likely due to the ubiquity of this interaction in terms of habitat – it is generally not limited to a particular environment, which differentiates it from other interactions in our dataset. As data on more and more interactions becomes available, we may begin to use such clustering techniques to gain more universal insight on the general global distributions of certain interaction types.

In addition, this analysis revealed proximity between some interactions based on mechanism of action. For example, siderophore-mediated signaling interactions reported separately by Lamont *et al*. in *Pseudomonas* ((Lamont *et al*. 2002), catalog entry 49) and Guan *et al*. in *Vibrio* ((Guan, Kanoh and Kamino 2001), catalog entry 73) are grouped within the same cluster. Interactions not mediated by any molecular exchange (Catalog entries 28, 32, 40, 41 and 46) are also largely grouped separately from those that require a molecular vehicle. In addition, observations of direct amino acid exchange ((Accolas, Veaux and Auclair 1971; Gobbetti, Corsetti and Rossi 1994; Holguin and Bashan 1996; Rikhvanov *et al*. 1999; Garcia *et al*. 2015), catalog entries 31, 12, 54, 55 and 9) display close proximity despite having been observed in very different habitats. This grouping shows that our clustering is sensitive to mechanism of action, and may provide a tool for identifying similar interactions across very different ecosystems.

Another feature of this multi-dimensional distance analysis is that it does not necessarily group interactions within the same benefit/detriment category together (e.g. all commensal interactions or all mutualistic interactions), as shown in the close groupings of a *Burkholderia*-*Rhizopus* toxin-dependent interaction (Partida-Martinez *et al*. 2007) and a eukaryote-bacteria protein-based relationship ((Schulz *et al*. 2016), catalog entries 17 and 18). These interactions, despite involving very different mechanisms, are similar in a less straightforward way: they are both mutualistic and both spatially and contact-dependent. Their close grouping therefore stems from this combination of features, which is rare in the dataset and places them at a greater distance from most other interactions. Such a use of a combination of factors to determine proximity is essential for more complete comparisons of interactions, and can also provide clues as to the types of interactions that may be rare in nature or have yet to be observed.

Lastly, this framework can be extended to analyzing interactions that involve more than two organisms. For example, the ‘ecological outcome’ and ‘cost’ categories can have as many columns as there are partners within any given interaction. In this way, it is possible to assign corresponding values to each participant within the entire multispecies relationship. To compare these attributes with those of other interactions, we used a pairing function to collapse these multivariable characteristics into single unique numerical values. In this way, for example, we can directly compare a synthetic tripartite symbiosis between *Azotobacter*, *Alternaria* and *Chlamydomonas* ((Lőrincz *et al*. 2010), catalog entry 8) with all others in our dataset. This example clustered very closely to a relationship between yeast and *Acinetobacter* ((Smith, Des Etages and Snyder 2004), catalog entry 52), allowing us to appreciate similarities in their mechanisms (both interactions involved the exchange of small molecules and have spatially dependent components) despite each having a different number of individual participants. If, however, an interaction involving more than two organisms can be divided into two clear roles, it is still possible to analyze it under a pairwise framework. For example, in the relationship involving *Thioploca*, a genus of marine sulfur bacteria, and a set of anaerobic ammonium oxidizing (anammox) bacteria, *Thioploca* is defined as the first participant as it was observed to unidirectionally provide small molecules to the anammox consortium, which is defined collectively as the second participant ((Scholten *et al*. 2007), catalog entry 68). We can then analyze such an interaction in the same way as we would those involving two distinct organisms. As a result, we notice that this interaction is grouped closely to other marine bacterial metabolic exchanges, showing that interactions with similar attributes can cluster together independently of the number of organisms involved.

The specific encoding structure we have proposed here is flexible, as different attributes can be highlighted using variants of the framework and refined as additional cases and data become available. In addition, our particular clustering analysis is highly sensitive to the distance metrics used and to the numerical inputs that were used to quantify individual attributes. Therefore, further research could address the question of whether alternative metric schemes should be used or different methods for dimensionality reduction could be employed. Regardless of the comparison techniques employed, however, we believe that a continued effort to formally encode interaction properties as we have done here could facilitate comparisons of diverse inter-microbial networks, especially as data from multiple microbial ecosystems are increasingly made available. While the formalization exemplified here is limited to a numerical representation of known attributes, a framework like it could help implement more comprehensive mathematical models for microbial ecosystem dynamics, applicable to understanding complex natural communities. For example, different interaction attributes could translate to specific terms in appropriate differential equations that describe community function (Carrara *et al*. 2015; Momeni, Xie and Shou 2017; Hart *et al*. 2019) enabling quantitative predictions of population dynamics. If available, data providing deeper information on interaction mechanisms, such as gene expression, binding affinities, and reaction rates could be also systematically encoded in future compendia. Such an encoding would need to be much more complex than the simple framework we have proposed here in order to enable comparison across interactions, but it would have the potential to facilitate quantitative community modeling efforts. For example, transporter parameters such as K_m_ and V_max_ values can be directly incorporated into metabolic cross-feeding simulations using genome-scale models (Khandelwal *et al*. 2013; Harcombe *et al*. 2014). Additionally, the design of synthetic microbial consortia could greatly benefit from the availability of a curated list of interaction properties. One could imagine selecting candidate organisms based on their known interactions in certain contexts, potentially expediting the process of assembling multispecies communities with desired phenotypes.

## SUMMARY

Descriptions of microbial interactions range from those that report individual symbioses in exquisite detail, to an increasing number of large-scale measurements of pairwise interactions enabled by new technologies. As we try to make sense of these interactions with the aid of network analyses and computer simulations, an emerging challenge is how to categorize these different types of relationships and formally encode their underlying mechanisms and attributes to enable comparison across datasets. In this mini-review, we provided examples of well-characterized microbial interactions to highlight their complex nature, and we illustrated the limitations of a broadly used ecological classification system. At the same time, we showed how one can in principle distill recurrent interaction characteristics that can be translated into multi-dimensional profiles. In an effort to embrace these details and characterize interactions in a more unified framework, we proposed a list of quantifiable attributes that can more fully capture the multidimensional nature of these phenomena. We compiled a collection of diverse interactions and quantitatively compared them according to these attributes, showing how such analysis can serve as a stepping stone towards more comprehensive quantitative frameworks for addressing important questions in microbial ecology.

As more data becomes available for a greater number of interactions, we may begin to incorporate additional attributes that can impact their interpretation, such as dependence on environmental substrates, pH or strain abundances. In addition, if non-mechanistic data from methods such as network inference are also to be incorporated into future iterations of this framework, it will be important to add identifiers to denote the experimental and computational techniques used to infer the resultant interactions. These considerations are crucial, as variability in different methods of inferring interactions is likely to have an impact on which data are available to collect and compare.

We anticipate that efforts similar to the one proposed here could grow into large databases of microbial relationships based on detailed observations, phenotypic measurements and ecosystem-level sequencing efforts. As we continue to gather data about microbial interactions and learn which of their attributes are most useful to encode, a standard format for these knowledge repositories (e.g. SBML for systems biology (Hucka *et al*. 2003) or SBOL for synthetic biology (Galdzicki *et al*. 2014)) may emerge to further codify reporting methods and facilitate comparative analyses. We therefore hope that, by compiling, classifying and analyzing this small collection of microbial relationships, our effort can motivate further efforts and conversations on how to gather, formalize and mine multi-dimensional data on microbial interactions.

## Supplementary Material

fnz125_Supplemental_File
